# Accelerating medical education: a survey of deans and program directors

**DOI:** 10.3402/meo.v21.31794

**Published:** 2016-06-13

**Authors:** Joan Cangiarella, Colleen Gillespie, Judy A. Shea, Gail Morrison, Steven B. Abramson

**Affiliations:** 1 Department of Pathology, New York University School of Medicine, New York, NY, USA; 2 Department of Medicine, New York University School of Medicine, New York, NY, USA; 3 Department of Medicine, Perelman School of Medicine, University of Pennsylvania, Philadelphia, PA, USA

**Keywords:** curriculum development, accelerated training, three-year MD

## Abstract

**Background:**

A handful of medical schools in the U.S. are awarding medical degrees after three years. While the number of three-year pathway programs is slowly increasing there is little data on the opinions of medical education leaders on the need for shortening training.

**Purpose:**

To survey deans and program directors (PDs) to understand the current status of 3-year medical degree programs and to elicit perceptions of the need for shortening medical school and the benefits and liabilities of 3-year pathway programs (3YPP).

**Methods:**

Online surveys were emailed to the academic deans of all U.S. medical schools and to a convenience sample of residency and fellowship PDs. Frequency distributions are reported for key survey items and content analysis was used to describe open-ended responses.

**Results:**

Of the respondents, 7% have a 3YPP, 4% were developing one, and 35% were considering development. In 2014, 47% of educational deans and 32% of PDs agreed that there may be a need to shorten medical school. From a list of benefits, both deans and PDs agreed that the greatest benefit to a 3YPP was debt reduction (68%). PDs and deans felt reduced readiness for independence, reduced exposure to complementary curricula regarding safety and quality improvement, premature commitment to a specialty, and burnout were all potential liabilities. From a list of concerns, PDs were concerned about depth of clinical exposure, direct patient care experience, ability to assume increased responsibility, level of maturity, and certainty regarding career choice.

**Conclusions:**

Over one-third of medical schools are considering the development of a 3YPP. While there may be benefits for a select group of students, concerns regarding maturity, depth of clinical exposure, and competency must be addressed for these programs to be well received.

The design of the majority of teaching programs in American medical schools has not changed since initially proposed by Flexner over 100 years ago (1) with 2 years of basic science followed by 2 years of clinical education. A number of medical schools have condensed the length of the basic science curriculum from the traditional 2-year curriculum to only 12 to 18 months which has allowed for earlier clinical exposures and led to discussion on whether the length of physician training could be shortened. This dialogue was further sparked by an article by Emanuel and Fuchs that suggested that overall medical training (college through fellowship) could be reduced by 30% without reducing competency of physicians (2). While two Canadian medical schools have been awarding medical degrees to their students after 3 years for decades, only a handful of medical schools in the United States award degrees after 3 years. However, calls for reform abound, including the 2010 Carnegie report which supports reforming the medical education system by enabling individualized learning and allowing students to progress after mastery of competency milestones (3). In such a model, a shortening of medical education would occur for those who achieve competency early as well as lengthening for those who take longer. As such, innovative, competency-based medical education models are in development (4). While the number of 3-year pathway programs (3YPP) is slowly increasing, there is little data on the opinions of medical education leaders on shortening training. In 2013, we surveyed educational deans (EDs) and residency program directors (PDs) to elicit their opinions and serve as the focus of an invited conference that ultimately led to a point–counterpoint article in the *New England Journal of Medicine*, describing differing views on accelerated pathway programs in medical education (5, 6). In 2014, a second survey was sent to deans and PDs to assess change in opinions on the shortening of training during medical school specifically and to explore in greater depth perceptions about the benefits and the liabilities of 3YPP. This paper describes the results of two national surveys of both undergraduate medical education (UME) and graduate medical education (GME) leaders in order to: 1) provide a snapshot of the status of 3YPP nationally; 2) explore changes in views on the need to shorten medical school training; and 3) identify the benefits, liabilities, selection criteria, and concerns associated with these programs.

## Methods

### Survey domains

The purpose of the first (2013) survey was to understand views on issues important to the future of physician training and included items assessing attitudes toward the shortening of physician training and when it should occur, and obstacles, benefits and strategies for shortening training. The second survey was designed to capture where medical schools were in terms of developing 3YPP and to assess views on the benefits, liabilities, selection criteria, and concerns about 3YPP. Individual items were created based on a review of the literature and questions about benefits and liabilities of 3YPP, selection criteria for students considering those pathways, and concerns about graduates were structured as a list of issues on which participants were asked to reflect and respondents were also given an opportunity to share other benefits, liabilities, criteria, and concerns through open-ended comments.

### Sample

Our sample consisted of deans of all U.S. medical schools for whom we could locate a valid email address and a convenience sample of residency PDs. For the sample of deans in 2013, we included only EDs (*n*=125 EDs); in 2014, we expanded our sample to include deans of medical schools (*n*=281 in total; *n*=125 medical school deans and 156 EDs). For the residency PDs’ sample, we were unable to obtain a complete listing of residency PDs and so used a convenience sample of PDs and email addresses that had been generated by the NYU alumni office for outreach to programs (*n*=3,150 in 2013 and *n*=3,094 in 2014). This sample size (*n*=3,499) and specialty distribution is similar to the National Residency Matching Program (NRMP) Directors’ Survey (7). However, Family Medicine programs are under-represented in our sample (5%) compared to the NRMP sample (17%), a likely reflection of the initial goal of the sample in reaching out to programs attended by our graduates since NYU medical school does not have a Family Medicine program.

### Survey methods

Individuals from this sample were sent an email with a link to a voluntary online survey. The email and introduction to the survey contained all the elements of written consent, and participants were considered to have consented if they chose to complete the survey. Email addresses were corrected as needed and survey invitations were re-sent whenever possible, leading to the identification of correct emails for all deans; however, approximately 5% of PDs had invalid email addresses that could not be corrected and therefore were dropped from the sample. Up to eight reminder emails were sent to non-respondents. The study was approved by the NYU School of Medicine's Institutional Review Board.

### Analyses

Analyses include frequency distributions for responses to survey questions and comparison of distributions of responses between 2013 and 2014 using chi-square test. Open-ended responses to targeted questions were coded for content and then the codes were grouped thematically, combining some low-frequency responses with related themes and dividing high-frequency responses into distinct sub-categories. JS created the initial coding and CG reviewed and both then discussed and reached consensus on the core coding scheme and its application to the open-ended responses.

## Results

### 2013 Survey

Sixty percent of EDs (75/125) responded. 56% (42/75) strongly or somewhat agreed that physician training could be shortened. 39% (29/75) felt the shortening could occur at the undergraduate level, 56% (42/75) during medical school, 29% (22/75) during residency, and 44% (33/75) during fellowship. 26% of PDs (810/3150) responded. 25% (203/810) of residency PDs felt training could be shortened. 51% (413/810) of PDs felt the shortening could occur at the undergraduate level, 29% (235/810) during medical school, 15% during residency (122/810), and 14% (113/810) during fellowship. Obstacles associated with shortening training included a lack of maturity (ED 40%; PD 58%), less of an academic focus (ED 34%, PD 59%), perceived competitiveness of graduates (ED 30%; PD 41%), and a loss of distinct professional identity (ED 24%; PD 36%). Similar percentages of deans and PDs endorsed the possible benefits associated with shortening training of early access to known career pathways (ED 65%, PD 61%) and accelerated pathways to research careers (ED 65%, PD 62%), but fewer PDs (compared to deans) endorsed the possible benefits of debt reduction (ED 89%; PD 80%), accelerated pathways to dual degrees (ED 71%, PD 60%), and eliminating the research requirement to focus on clinical practice with competency-based milestones (ED 64%, PD 58%).

Of the EDs, 45% (34/75) felt that residency programs at their institution would accept applicants from 3YPP. If there was to be shortening of medical school, the majority (84%) felt training could be shortened through competency-based progression and should be permitted for only a subset of students (84%) (63/75). Of the residency PDs, 66% (535/810) felt that the program length for their specialty was optimal and 47% (381/810) indicated that they would accept applicants from 3YPP.

### 2014 Survey

Response rates were 55% for deans (154/281). Of the 154 deans that responded, 67 were EDs and 87 were deans of medical schools (D). 28% of PDs (880/3,094) responded. 71% (48/67) of EDs felt physician training could be shortened (a substantial but not significant increase over 2013s 56%; *χ*^2^=4.26, *p*=0.255); with 47% (31/67) agreeing with the need to shorten medical school (also an increase over the 2013 percent of 56%, albeit not a significant change; *χ*^2^=4.21, *p*=0.240). 42% (370/880) of residency PDs felt physician training could be shortened with 32% agreeing with the need to shorten medical school. The need to shorten medical school showed a slight increase compared to 2013 (29%) in overall agreement (*χ*^2^=26.67, *p*<0.001) of PDs.

Of the 127 medical schools represented in this sample (based on having at least one survey respondent from that medical school), 7% have a 3-year pathway, 4% are developing one, and 35% are considering development (Table 1). The majority of schools do not expect a 3-year pathway to be offered to the entire class. 76% of those schools who have or are planning programs expect to have less than 25% of the class enroll in a 3-year pathway; 50% expect less than 10%.

**Table 1:** Status of medical schools regarding 3-year pathway programs (deans’ views, *n*=127)

Accelerated pathway status	% of schools	Description/examples if implemented, developing or considering accelerated pathway
Already have	7% (*n*=9)	In 5 schools, pathway is for <10% of students In 3 schools, pathway is for up to 50% of students 1 school is entirely a 3-year program
In development	4% (*n*=6)	Examples: Tied to specific residency programs (e.g., primary care or family medicine) Combined with 3-year pre-medical curriculum as part of a multi-school state initiative Integration of UME and GME
Considering developing accelerated pathway	35% (*n*=44)	Examples Fourth year would be self-directed career development Use EPAs and competence-based tools to accelerate graduation for qualifying students Tied to specific residency programs (e.g., primary care or family residency) as part of longitudinal integrated clerkship pathway Self-paced curriculum PhD to MD model Accelerated basic sciences including summer between MS 1 and 2 Fourth year becoming first year of residency for some disciplines
Rejected accelerated pathway (considered but rejected)	17% (*n*=21)	N/A
Have not yet considered	34% (*n*=43)	
Did not respond	3% (*n*=4)	

### Benefits

**Fig. 1:**
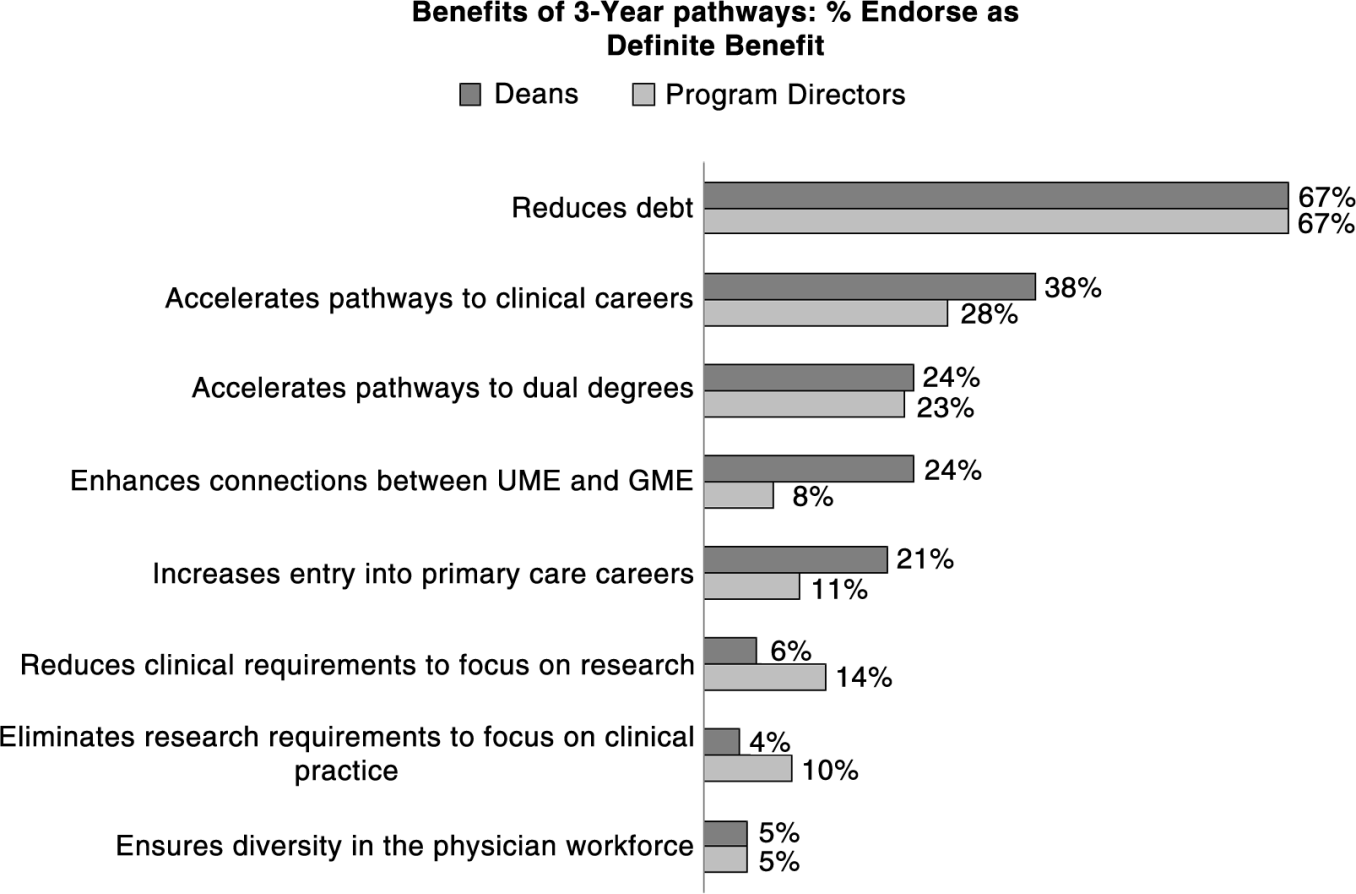
Perceived benefits of 3-year pathway programs (2014 Survey; 154 deans and 880 PDs). Survey item: To what extent do you agree that each of the following is a potential benefit of the 3-year pathway? Response options: not a benefit at all; only a little bit of a benefit; somewhat of a benefit; definitely a Benefit. Percentages reported in the figure are for number of respondents who chose *Definitely a Benefit*.

In responding to a list of possible benefits (Fig. 1), the largest percent of both deans and residency PDs (67%) endorsed debt reduction as a benefit. Accelerating pathways to clinical careers was endorsed by about a third (38% of deans and 28% of PDs). Enhancing connections between UME and GME was endorsed by 24% of deans but only 8% of PDs, and similarly, increasing pathways to primary care careers was endorsed by 21% of deans and 11% of PDs. Reducing clinical requirements to focus on research careers eliminating research requirements to focus on clinical practice, and ensuring diversity in the workforce were perceived by fewer respondents as a benefit.

Benefits most commonly reported in response to an open-ended prompt included restatement of the reduction of debt burden (e.g., ‘Debt burden is the primary factor. It reduces the number of candidates for primary and non-procedural specialties’.). The other common responses involved elaborations on the benefit of getting physicians into the workforce earlier (e.g., ‘More efficient training pathways in the context of enrolling mature individuals with prior exposure and commitment to clinical care’.).

### Liabilities

**Fig. 2:**
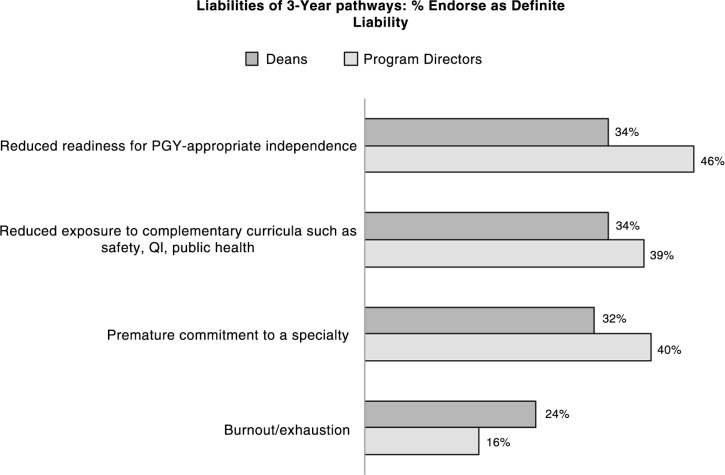
Perceived liabilities of 3-year pathway programs (2014 Survey; 154 deans and 880 PDs). Survey item: To what extent do you agree that each of the following is a potential liability of the 3-year pathway? Response options: not a liability at all; only a little bit of a liability; somewhat of a liability; definitely a liability. Percentages reported in the figure are for number of respondents who chose *Definitely a Liability*.

Of the list of four liabilities shown in Fig. 2, none were endorsed by more than half of respondents but residency PDs tended to endorse at higher rates than deans. Close to half of PDs saw reduced readiness for PGY-appropriate independence as a liability, whereas only about a third of deans endorsed as a definite liability. PDs also seemed slightly more concerned about premature commitment to a specialty but less concerned about burnout/exhaustion than deans (16% of PDs vs. 24% of deans cited as a definite liability). Responses to the opportunity to describe other liabilities focused on: 1) less competent physicians (e.g., ‘Poor understanding of medicine in general. Reduced overall competence’, ‘It is already very difficult for students to learn the body of knowledge to be a good physician’, ‘My opinion of the knowledge base for incoming interns for basic pathophysiology and disease processes is very low already for the 4 year students’.); 2) decreased time to explore other specialties and make career choices (e.g., ‘Working with 3rd year students now, there is tremendous anxiety about career choice. I've seen students change directions completely after a 4th year elective or feel much more comfortable about their career choice’ and ‘Reduced exposure to and proficiency in multiple medical specialties; less likelihood to enroll in physician/scientist pathways’); 3) further concerns about maturity and personal development (‘Lack of maturity in dealing with human beings’ suffering, pain needs, in relating to patients, empathizing with patients’, ‘emotional maturity’, ‘clinical maturity’); and 4) specific losses to the curriculum (e.g., ‘content – safety, QI, research experience’, ‘something has to give’, reduced clinical exposure and exposure to ‘science’).

Open-ended responses focused on competency and producing physicians that were less experienced, knowledgeable, and well rounded. Other liabilities included less time to explore other specialties and less time to make a career choice. Maturity and the inability to teach critical pieces of curriculum, i.e., patient safety, communication skills, were also noted liabilities.

### Selection criteria

Of the selection criteria provided to respondents, the top three criteria for selecting 3-year pathway students that were rated as somewhat or very important by most PDs were maturity (88%), prior clinical experience (60%), and a high GPA (60%). Other criteria were also cited as being important by a substantial percent of PDs (55% felt a high MCAT score was important, 46% a prior gap year, and 45% felt prior research experience was important).

PDs contributed 193 open-ended responses regarding criteria for the selection of students. Sixty-five were focused on career plans (e.g., ‘students who already know exactly what field of medicine they plan to pursue and are most likely not going to need the extra time to figure it out’, ‘if a student knew they were going to be a scientist and didn't need all that clinical training or if the student knew they were going into primary care, for the same reason’, ‘desire to enter a specialty with a long GME training period’). Thirty responses focused on prior experience (e.g., ‘Past history demonstrating intelligence, ability to balance education/study with other activities – work, sports which demonstrates that they have not been successful only at book learning and test taking’. ‘Work and leadership experience – how well and in what environments have they worked with people effectively (teams, project management, service delivery)’, ‘Emotional intelligence – self-awareness, self-management, social awareness and relationship management are key’). Twenty-five responses centered around the motivation of a student to enter a 3-year pathway including reasons for wanting to become a physician, commitment to practicing medicine as well as direct motivations for wanting to complete quickly, e.g., reducing debt versus ‘using the year in an alternative manner for gaining expertise’. Twenty-five responses included recommendations to select students with prior degrees (PhDs or degrees in other healthcare fields). Twenty-four comments related to a student's undergraduate focus (with the overwhelming majority looking for extensive basic science coursework) and 11 on restatements of the importance of maturity. These concerns around maturity were described in three broad areas: 1) emotional, social, and personal development; 2) life experiences and responsibilities; and 3) time for knowledge, clinical skills, and judgment to develop into a solid foundation for clinical decision-making.

### Concerns

**Fig. 3:**
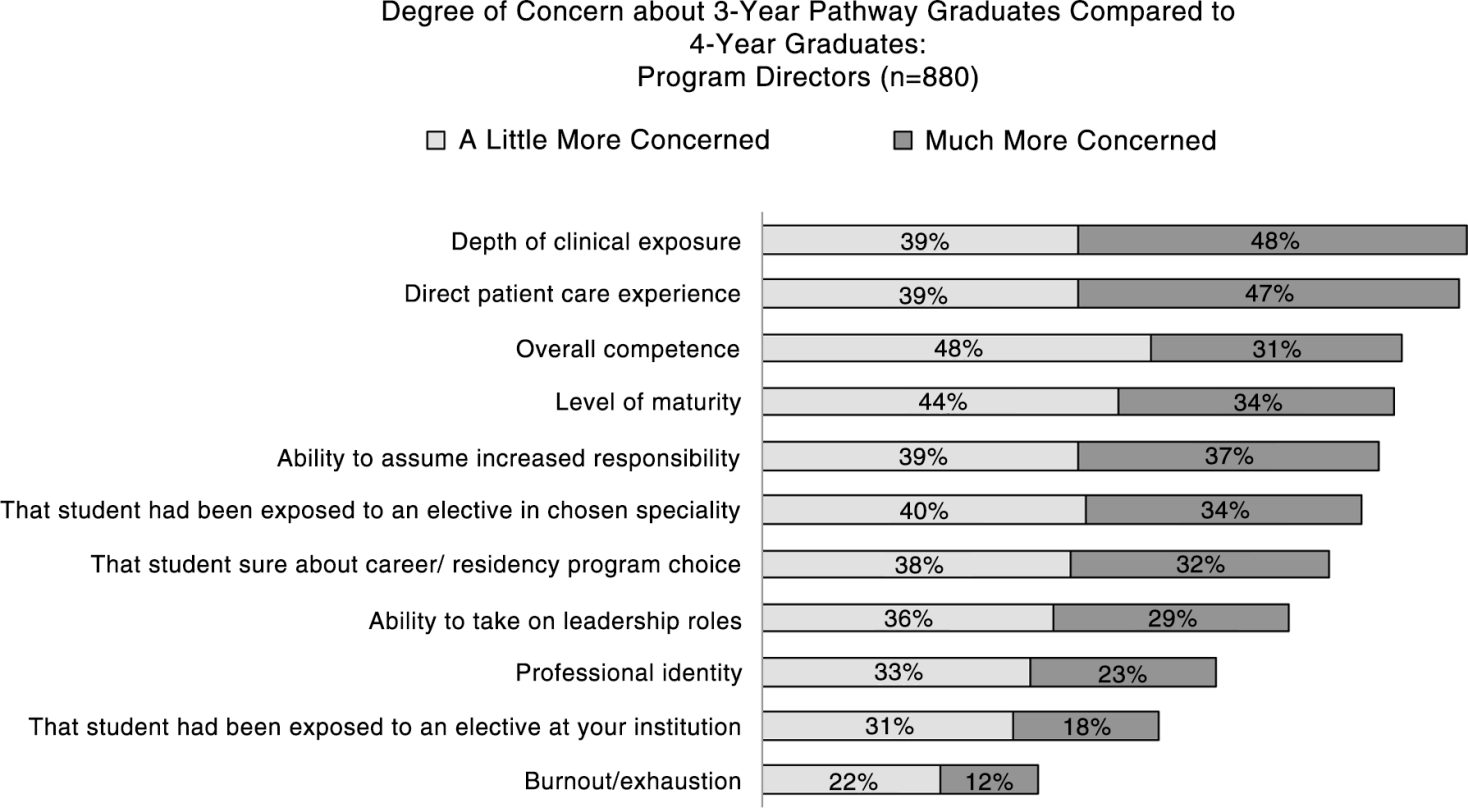
PDs concerns regarding 3-year pathway graduates compared to 4-year graduates.

Of a list of concerns provided in Fig. 3, PDs were most concerned about 3-year graduates in terms of their depth of clinical exposure, direct patient care experience, ability to assume increased responsibility, level of maturity, and certainty regarding career choice (Fig. 3). Of the open-ended comments among programs, directors who would likely not accept a 3-year pathway student concerns related to immaturity (*n*=48), insufficient clinical exposure (*n*=45), and level of certainty for specialty choice (*n*=19). For those PDs who would likely accept a 3-year pathway student, the great majority indicated in the open-ended responses that they would judge a student with a strong portfolio on a case by case basis (e.g., ‘We would review each applicant case by case, recognizing that some individuals will be very successful with only 3 years while others should have 4 years’, ‘Smart, talented, and well-trained people happen through a variety of pathways. Although I have serious concerns about the acceleration of an already difficult journey I am not willing to summarily close off paths into our residency program without evaluating such a candidate on her or his merits’). Some respondents pointed to the curriculum as essential for making this determination: ‘The acceptance would very much depend on the 3 year curriculum. We would accept graduates who have had in depth exposure to patient care – face to face, and have been exposed to a rigorous curriculum in communication skills’. Best suited students would be mature, have life experiences, and a commitment to their specialty choice, and acceptable curriculum would be rigorous and designed with sufficient focus on core clinical skills.

Even with their concerns, 49% of residency PDs and 45% of deans felt that the residency programs at their own institutions would accept 3-year pathway students while only 34% reported that residency programs in general would accept 3-year pathway students.

Of a list of components that should be included in the design of a 3YPP, the vast majority of PDs felt clear criteria for completion (90%) and monitoring of performance (89%) were very important components of the design; clear criteria for admissions, mentoring, and flexible opt-in/opt-out options were also cited as important by a majority of PDs.

## Discussion

A recent *Wall Street Journal* article (8) describes the shortening of the 4-year curriculum to 3 years as one of several innovations currently impacting medical school education. Supporters of 3YPP highlight the desirability of individualized pathways for learners with diverse backgrounds and interests, the need to reduce the financial burden of medical school, the need to address the physician shortage, and also the ability to enable linkage between UME and GME (5). Joining UME with GME training advances a new training paradigm that unfolds over 7 (or more) years. Across this UME–GME continuum students receive intense mentorship, integration into departments through education (shadowing, attendance at grand rounds), and research opportunities. The UME–GME continuum model allows the tracking of student's performance and development. Accelerated programs give students choice in individualizing their coursework and for those who were certain about their career choice an ability to accelerate their careers, reduce debt, and participate in research and education in the field they were most interested in.

Opponents of 3YPP cite student exhaustion, faculty dissatisfaction with condensed curriculum content, and the lack of education in global health, ethics, and health policy. If the fourth year is eliminated, benefits that include time to master skills learned during the clerkship year and time to narrow down career choices are lost (6).

Both supporters and opponents of 3YPP recognize the significant challenges. The concerns include reduced readiness for independence, depth of clinical exposure, overall competence, and maturity of accelerated students. While opponents to 3YPP cite competency as a main concern, conclusive data that the length of curriculum is related to student, resident, or physician performance is lacking. Comparison of medical schools in Alberta, Canada, showed equivalent performance of graduates from 3- to 4-year pathways (9). For accelerated programs to be successful, enrollees must be as prepared as their 4-year counterparts to perform successfully in residency training programs. As noted within the survey, while more than half of the residency PDs would be willing to accept students from a 3-year pathway program they described the most important components of the design of 3YPP to include monitoring of performance, clear criteria for completion and admission, mentoring, and the ability to opt-out of an accelerated pathway program. A focus on measuring and demonstrating competency will be essential. This is especially true if an accelerated pathway student is to be a successful applicant to residency programs at institutions other than their own. Finally, it is critical to nurture and maintain the professional identity of these future physicians throughout their training. This will be accomplished not only by ensuring competency of accelerated program graduates but also by conceptualizing training as a journey of 7–10 years (or longer if premedical education is included).

The debate over 3-year pathways has also led to increased focus on the fourth year and understanding how the fourth year can be made more meaningful in preparing students in their transition to residency. It is important to note that most medical schools interested in developing 3-year pathways intend to do so as a track for a minority of the class (e.g., 10–25%), and therefore, the importance of the fourth year is relevant to schools with or without fast-tracks. As compared to the first 3 years of medical school education, the fourth year is largely unstructured but viewed by some as a time to define career goals. The majority of the fourth year in most schools continues to be composed of electives, with an average of 22.3 weeks in a 36-week curriculum (10). The consequences of developing a burdensome residency application process that requires reliance on audition interviews by applicants and an unlimited time span for interviews has reduced the amount of meaningful educational activities in the fourth year (11). Concerns regarding the fourth year highlighted in a survey in 2000 included a lack of understanding of the educational purpose of the fourth year, problems in the design and content of the curriculum, and concerns with educational quality (10).

Understanding student views on the fourth year is meaningful. One study showed that while students described the purpose of the fourth year to strengthen their residency applications, to develop skills required for residency, to pursue personal interests, and to explore diverse practice settings that would ultimately lead to the identification of a career, over 57% felt the purpose was to take time off and have more time for themselves (12). Another study that looked at residents’ views of the fourth year of medical school (13) proposed a framework that would allow solidification of educational goals while maintaining individualized pathways. This framework offered three recommendations that included specification of the required competencies and milestones required for graduation, individualized learning plans, and a support structure to assure the achievement of the required outcomes and personal goals during the fourth year. This framework can be easily applied to the success of an accelerated pathway program.

While our two surveys indicate that only 7% of medical schools currently have a 3YPP, over one-third of medical schools are considering the development of such programs. Shortening of medical school has emerged as a possibility for a specific subset of students. Competency-based programs that are time variable are also developing. It is clear that the success of 3YPP in medical school will only succeed if such programs can assure the competencies, maturity, professionalism, and attractiveness of these graduates.

## References

[1] Flexner A (1910). Medical education in the United States and Canada: a report to the Carnegie Foundation for the Advancement of Teaching.

[2] Emanuel EJ, Fuchs VR (2012). Shortening medical training by 30%. JAMA.

[3] Irby DM, Cooke M, O'Brien BC (2010). Calls for reform of medical education by the Carnegie Foundation for the Advancement of Teaching: 1910 and 2010. Acad Med.

[4] Powell DE, Carraccio C, Aschenbrener CA (2011). Pediatrics redesign project: a pilot implementing competency-based education across the continuum. Acad Med.

[5] Abramson SA, Jacob D, Rosenfeld M, Buckvar-Keltz L, Harnik V, Francois F (2013). A 3-year M.D. – accelerating careers, diminishing debt. N Engl J Med.

[6] Goldfarb S, Morrison G (2013). The 3-year medical school – change or shortchange?. N Engl J Med.

[7] National Resident Matching Program (2014). Data Release and Research Committee: results of the 2014 NRMP Program Director Survey.

[8] Beck M (2015). Innovation is sweeping through U.S. medical schools. Wall Street J.

[9] Lockyer JM, Violato C, Wright BJ (2009). An analysis of long-term outcomes of the impact of curriculum: a comparison of the three- and four-year medical school curricula. Acad Med.

[10] Barzansky B, Simon FA, Brotherton SE (2001). The fourth-year medical curriculum: has anything changed in 20 years?. Acad Med.

[11] Aagard EM, Abaza A (2016). The residency application process – burden and consequences. N Engl J Med.

[12] Wolf SJ, Lockspeiser TM, Gong J, Guiton G (2014). Students’ perspectives on the fourth year of medical school: a mixed-methods analysis. Acad Med.

[13] O'Brien B, Niehaus B, Teherani A, Young J (2012). Residents’ perspectives on the final year of medical school. Intern J Med Educ.

